# Prophylactic and postoperative antibiotic therapy for coronectomy procedures in mandibular third molars: mapping the evidence through a scoping review

**DOI:** 10.1007/s10006-026-01516-w

**Published:** 2026-03-06

**Authors:** Lucas Jardim da Silva, Laura Lourenço Morel, Júlia Rodrigues Burkert, Josué Martos, Cristina Braga Xavier, Melissa Feres Damian

**Affiliations:** 1 Post-Graduate Program in Dentistry, Federal University of Pelotas, Pelotas, Rio Grande do Sul Brazil; 2 Pelotas, Rio Grande do Sul Brazil; 3 Department of Clinical and Diagnostic Sciences, Federal University of Pelotas, Pelotas, Rio Grande do Sul Brazil; 4 Department of Oral and Maxillofacial Surgery and Traumatology, Federal University of Pelotas, Pelotas, Rio Grande do Sul Brazil

**Keywords:** Oral surgical procedure, Third molar, Antibiotic, Review literature as a topic

## Abstract

**Purpose:**

This scoping review systematically maps and summarizes the available evidence, identifies patterns, and highlights gaps in the current literature on antibiotic use in coronectomy procedures.

**Methods:**

Databases were searched up to June 2025 for human clinical studies evaluating the coronectomy technique for mandibular third molars that reported pre- or postoperative antibiotic use. In addition to manuscript identification, study design, evaluated outcomes, and sample characteristics, the collected data focused on antibiotic regimens, postoperative infection rates, and other reported complications, such as dry socket. The data were analyzed descriptively and summarized via tables and figures.

**Results:**

Fifty studies evaluating approximately 2,126 patients were included. The included studies reported substantial heterogeneity in antibiotic protocols for coronectomy. Postoperative antibiotic administration was the most frequently described approach, most commonly involving amoxicillin, either alone or in combination with clavulanate for 3–7 days, whereas other agents were prescribed less frequently. Postoperative infection and complications were reported in approximately 60 patients (2.82% of patients sample). Amoxicillin was the most frequently reported antibiotic in these cases, general practice reflecting its overall predominance in coronectomy protocols rather than indicating any association with postoperative complications.

**Conclusion:**

The available evidence remains insufficient to support standardized antibiotic protocols or to determine the necessity of routine antibiotic use in coronectomy procedures. These findings underscore the importance of cautious antibiotic prescribing and highlight the need for further controlled studies to better inform clinical decision-making.

**Supplementary Information:**

The online version contains supplementary material available at 10.1007/s10006-026-01516-w.

## Introduction

Approximately 40% of the mandibular third molars fail to erupt functionally into the oral cavity, remaining partially or completely impacted within the bone [1]. Consequently, the surgical extraction of these teeth represents one of the most frequent performed procedures in oral surgery, accounting for around 35.9% to 58.7% of interventions in specialized services [1, 2]. Although most complications associated with the surgical removal of mandibular third molars are self-limiting, neurosensory injuries, particularly those involving the inferior alveolar nerve (IAN), are clinically significant and may negatively affect patients’ quality of life [1, 3, 4]. These disturbances, which may manifest as hypoesthesia, paresthesia, or dysesthesia of the lower lip, gingiva, or chin, have been reported with an incidence ranging from 0.4% to 8% in temporary cases, whereas permanent disturbances are reported in approximately 1% to 3% of cases [2, 3]. However, in situations where the roots of impacted third molars are in close proximity to the IAN, the frequency of neurosensory disturbances may reach up to 35% [4].

These data underscore the clinical relevance of alternative surgical approaches aimed at reducing the risk of neurosensory injury when removal of impacted mandibular third molars is indicated. Among these alternative approaches, coronectomy, or intentional partial odontectomy, has been proposed [5], with clinical and scientific [4, 6, 7]. This technique consists of removing completely the dental crown and intentionally retaining the roots of impacted mandibular third molars within the alveolar socket [8, 9]. In recent meta-analyses [2, 4], coronectomy was associated with consistent reduction in the risk of IAN injury, when compared with conventional extraction.

In addition to neurosensory disturbance involving the IAN, comparisons between coronectomy and conventional extraction have also been explored with respect to other postoperative outcomes [3, 4, 9]. Overall, the literature indicates that coronectomy is associated with a lower incidence of dry socket or localized alveolitis when compared with total odontectomy, whereas findings related to postoperative pain and swelling remain variable and methodologically heterogeneous [2, 4]. Conversely, coronectomy presents a distinct profile of late events, such as root migration and root exposure, which are more frequently associated with an increased need for surgical reintervention [3, 9]. Regarding postoperative infection, current evidence suggests that infection rates following coronectomy are not consistently lower than those observed after complete third molar extraction. Rather, reported infection rates appears to be influenced by operative- and case-related factors, including surgical time, surgeon experience, patients’ comorbidities, and antibiotic use, with substantial heterogeneity across studies [2, 4, 6].

In this context of heterogeneous and inconclusive evidence regarding postoperative infection outcomes, the role of antibiotic therapy remains controversial. Recent studies have reported substantial variability in prescribing practices, ranging from universal prophylaxis protocols [3, 10] to exclusively therapeutic use in selected situations, such as active infection, systemic risk, or complex surgery procedures [1, 11]. This variability appears to be influenced by the absence of standardized clinical guidelines, differences in surgeon preference, and variability in the perceived risk of postoperative infection across clinical scenarios [6].

Clarifying this issue is essential to support evidence-based clinical decision-making, particularly in the context of growing concerns about antimicrobial resistance and the need for prudent antibiotic prescribing. However, the available evidence on antibiotic use in coronectomy procedures is heterogeneous in terms of study design, clinical indications, outcomes reporting, and prescribing protocols, which precludes meaningful quantitative synthesis. Therefore, a scoping review was conducted to systematically map and summarize the available evidence, identify patterns, and highlight gaps in the current literature on antibiotic use in coronectomy procedures.

## Materials and methods

### Study design

This scoping review was conducted following the PRISMA/ScR (Preferred Reporting Items for Systematic Reviews and Meta-analyses extensions for Scoping Reviews) [12] and the Joanna Briggs Institute methodological guidelines [13].

### Protocol

The project was registered in the Open Science Framework (OSF) database (DOI: 10.17605/OSF.IO/WA8CZ) to ensure transparency and methodological rigor. The registration included the study protocol, objectives, and data extraction strategy.

### Review question

The review question, structured according to the PCC (population, concept, and context) framework, was as follows: *“In patients undergoing coronectomy*,* under which clinical conditions are antibiotics indicated*,* and what are the commonly prescribed agents*,* dosages*,* and durations of administration?”*

### Search methods

**Fig. 1:**
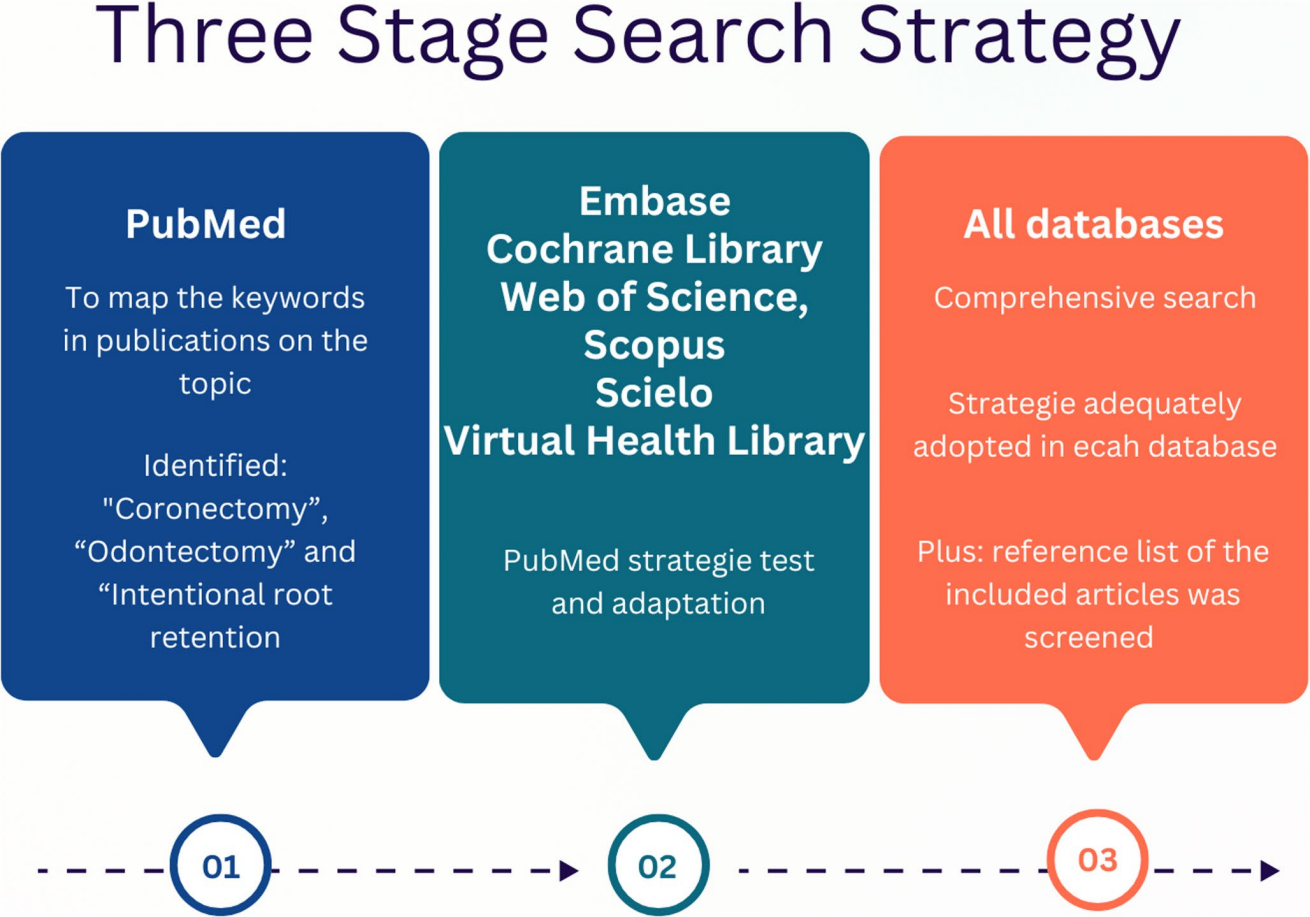
Three-stage search strategy adopted for the scoping review, according to Prisma/ScR

A three-stage search was conducted (Fig. 1) individually by three trained researchers (LJS, LLM, and JRB), with disagreements solved by consensus with senior investigators (MFD and CBX). The detailed database search is provided in Supplementary Table 1.

### Study selection

The records retrieved from each database were imported to the Mendeley Desktop™ reference manager (Mendeley Ltda, Relx Group™ Elsevier, London, UK). After removing the duplicates, a consolidated file containing all identified studies was exported into the Rayyan application [14] for the selection process. Screening was performed independently by the three investigators responsible for the search in two stages: (1) title and abstract screening and (2) full-text review. In the second stage, the reasons for exclusion were recorded. Any disagreements were resolved by consensus with the involvement of the senior investigators. The inclusion and exclusion criteria for study selection are described in Box 1.

Box 1: Inclusion and exclusion criteria established for study selection

**Table:** 

	Criteria
Included	1. Studies evaluating or describing the coronectomy technique for mandibular third molars
	2. Studies reporting the use of antibiotics in coronectomy procedure, either preoperatively or postoperatively
	3. Clinical studies (randomized or nonrandomized) or observational studies, as well as case reports or case series
	4. Studies conducted in humans, classified as ASA I to II [15]
Excluded	1. Inability to obtain information on antibiotic prescription or absence of antibiotic prescription associated with coronectomy
	2. In vivo or in vitro studies, reviews, editorials, letters to the editor, and conferences abstracts
	3. Coronectomy procedure in tooth other than mandibular third molars
	4. Unintentional coronectomy (transoperative accidents)
	5. Studies with duplicate samples (in which case, it was included the study with largest sample)
	6. Studies involving patients classified as ASA III to VI [15]

Eligibility criteria were defined a priori to align with the objective of a scoping review, focusing on mapping the extent and characteristics of evidence on antibiotic use in coronectomy procedure rather than estimating comparative effects. Case reports and case series were considered eligible because they may contribute relevant descriptive information on prescribing practices, clinical indications, and postoperative management in a literature base that is heterogeneous and includes limited high-level evidence. Restricting inclusion to patients classified as ASA I–II was intended to improve clinical comparability and to reduce confounding related to systemic conditions that could independently influence postoperative outcomes, infection risk, and antibiotic prescribing. In addition, studies in which antibiotics were not prescribed were excluded because the scope of the review was specifically to characterize antibiotic use in coronectomy procedures.

### Data extraction

Data extraction from the included articles was performed independently by the same three researchers involved in the previous stages and verified by consensus with the senior authors, who also solved any disagreements. The data were recorded in a Microsoft Excel™ spreadsheet (version 16.0, Redmond, WA, USA), which was specifically designed for the study on the basis of the Joanna Briggs Institute’s data extraction form [13]. This extraction tool was tested with five randomly selected articles (10% of the sample) to assess its adequacy and determine the need for adjustments.

The following information was extracted from the primary studies: (1) manuscript identification (authors, year of publication, country of origin and language); (2) study design; (3) evaluated outcomes; (4) sample characteristics [number of participants (total and by sex); age (range and mean ± standard deviation for clinical and observational studies and case series; individual for case reports); number of teeth undergoing coronectomy (overall and by side: #38 or #48)]; (5) adjuvant postoperative therapy (presence, type and number of patients treated); (6) preoperative pathologies associated with the mandibular third molars (presence, type and number of patients affected); (7) antibiotics used (drug, dosage and posology); (8) timing of administration (preoperative, postoperative, or both); (9) immediate postoperative infection rates (number of patients or teeth affected); (10) late postoperative infection rates (number of patients or teeth affected and follow-up period occurrence); 11) other postoperative complications related to the use of antibiotics (type, number of affected patients or teeth, and the period of occurrence); and 12) how long the patients were followed.

Immediate infection rates were defined as those occurring until seven days post-operatively, typically corresponding to the period before suture removal, whereas late infection rates were those occurring thereafter. Events such as alveolitis or dry socket were recorded as “other postoperative complications related to the use of antibiotics”. Complications related to coronectomy, such as root migration, or events commonly expected in a dental surgical procedure, such as swelling, were not recorded, as they are not directly related to infectious outcomes and were inconsistently reported across studies, limiting their relevance to the objective of mapping antibiotic use. When the data were unclear, the study authors were contacted via email or social media.

### Data analysis

The information collected from the studies was organized into tables and figures to provide a clear representation of the results. The tables were created via Microsoft Word™ (version 16.0, Redmond, WA, USA), whereas the figures were generated via the Flourish Studio platform (Canva UK Operations Ltd, London, UK), which is available at www.flourish.studio.

## Results

### Search

**Fig. 2:**
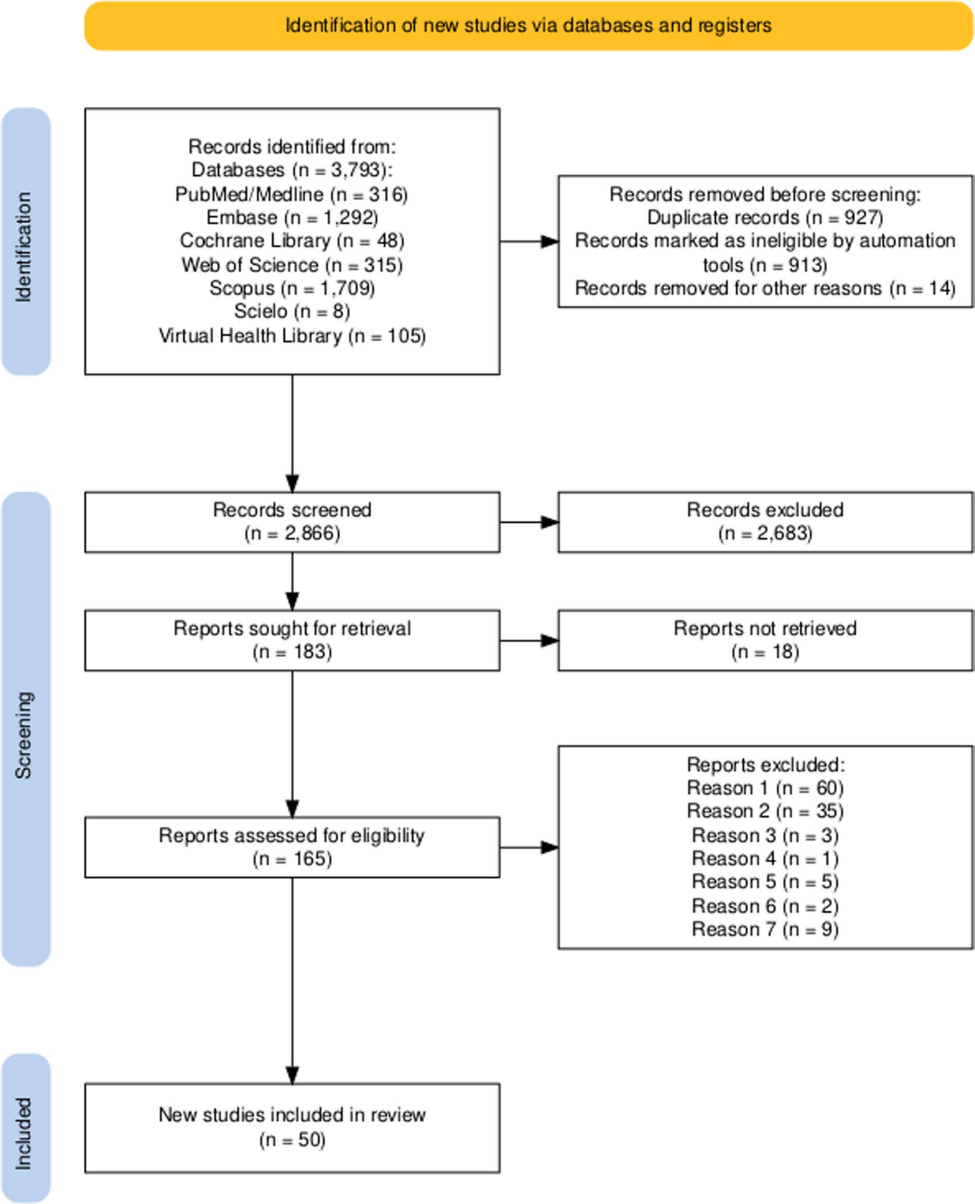
Flow diagram of the study selection process [designed using the Evidence Synthesis Hackathon (ESH) platform]. The reasons for exclusion were: (1) absence of antibiotic prescription or insufficient information on antibiotic use; (2) study design; (3) teeth other than mandibular third molars; (4) unintentional coronectomy; (5) duplicate samples; (6) inclusion of patients classified as ASA III to VI; and (7) other reasons (conventional odontectomy, exclusively radiographic follow-up data, language, ostectomy technique, patient refusal of coronectomy, and treatment of pericoronitis)

The search was conducted up to June 2025, yielding 3,793 references (PubMed/Medline = 316; Embase = 1,292; Cochrane Library = 48; Web of Science = 315; Scopus = 1,709; SciELO = 8; Virtual Health Library = 105). These records were exported to the Mendeley reference manager, where 913 duplicates were removed, resulting in a consolidated file of 2,880 references. This file was imported into the Rayyan application for study selection, where an additional 14 duplicates were identified and removed, leaving 2,866 references for screening. In the first selection step (title and abstract screening), 2,683 records were excluded, with a mean agreement of 91.65% between reviewers, leaving 183 articles for full-text assessment. In the second step (mean agreement = 72.22%), 18 references could not be retrieved (despite attempts to contact authors and search library holdings), resulting in 165 full texts assessed for eligibility. Among these studies, 115 were excluded (reasons listed in Supplementary Table 2), leaving 50 studies that met the inclusion criteria. The complete screening process is illustrated in Fig. 2.

### Characteristics of the included studies

Most of the primary studies included in this scope review reported following coronectomy and/or conventional mandibular third molar extraction, with emphasis on postoperative outcomes, particularly those related to inferior alveolar nerve injuries. Remnant root migration and its clinical implications were also frequently described. Notably, only one study specifically evaluated the use of antibiotics in coronectomy [1]: a retrospective observational study published in 2025 by Norwegian researchers, which focused on postoperative complications related to the procedure (Supplementary Table 3). The remaining 49 articles mentioned antibiotics only in the description of the surgical procedure, within the “Materials and Methods” or “Case Report” sections.

**Fig. 3:**
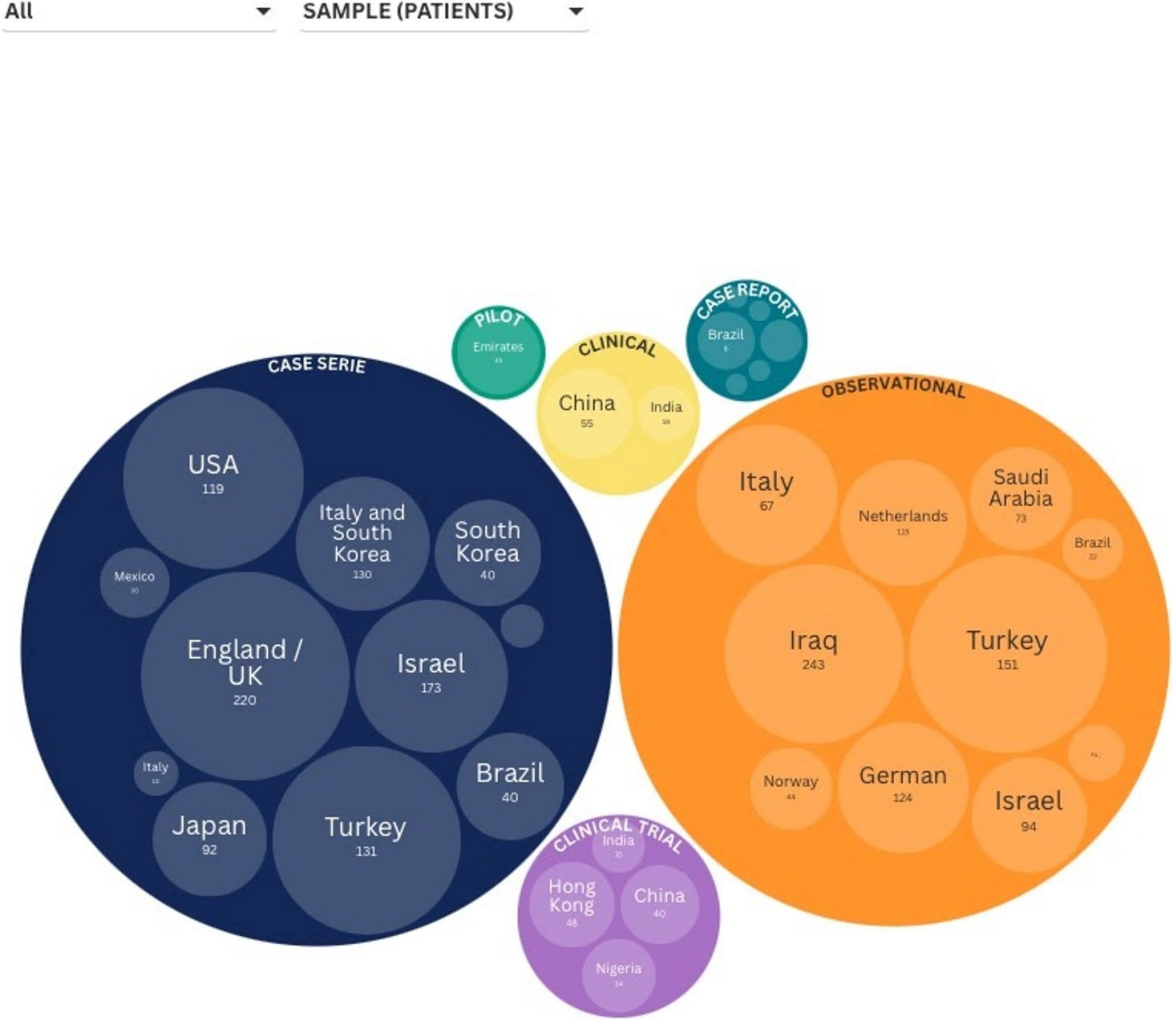
Characteristics of the included studies. An interactive version of these grouped circle graphics is available online at https://public.flourish.studio/visualisation/24724904/. The interactive version allows users to further explore the data. Each graphic represents the individual visualization of the sample (patients and teeth) according to the different primary study designs. Created using Flourish (https://flourish.studio)

Case series and observational studies were the most prevalent methodological approaches, encompassing the majority of the approximately 2,126 patients and 2,129 mandibular third molars evaluated. Case reports were also prevalent among the included studies; however, owing to their design, they contribute a limited number of patients and teeth to the overall sample. The vast majority of all the articles were published in English, although their countries of origin varied considerably. The reported follow-up periods ranged from 1.5 months [16] to 10 years [17–19] (Fig. 3, Supplementary Tables 3 and 4 show the extracted data in detail).

### Main data collected

**Fig. 4:**
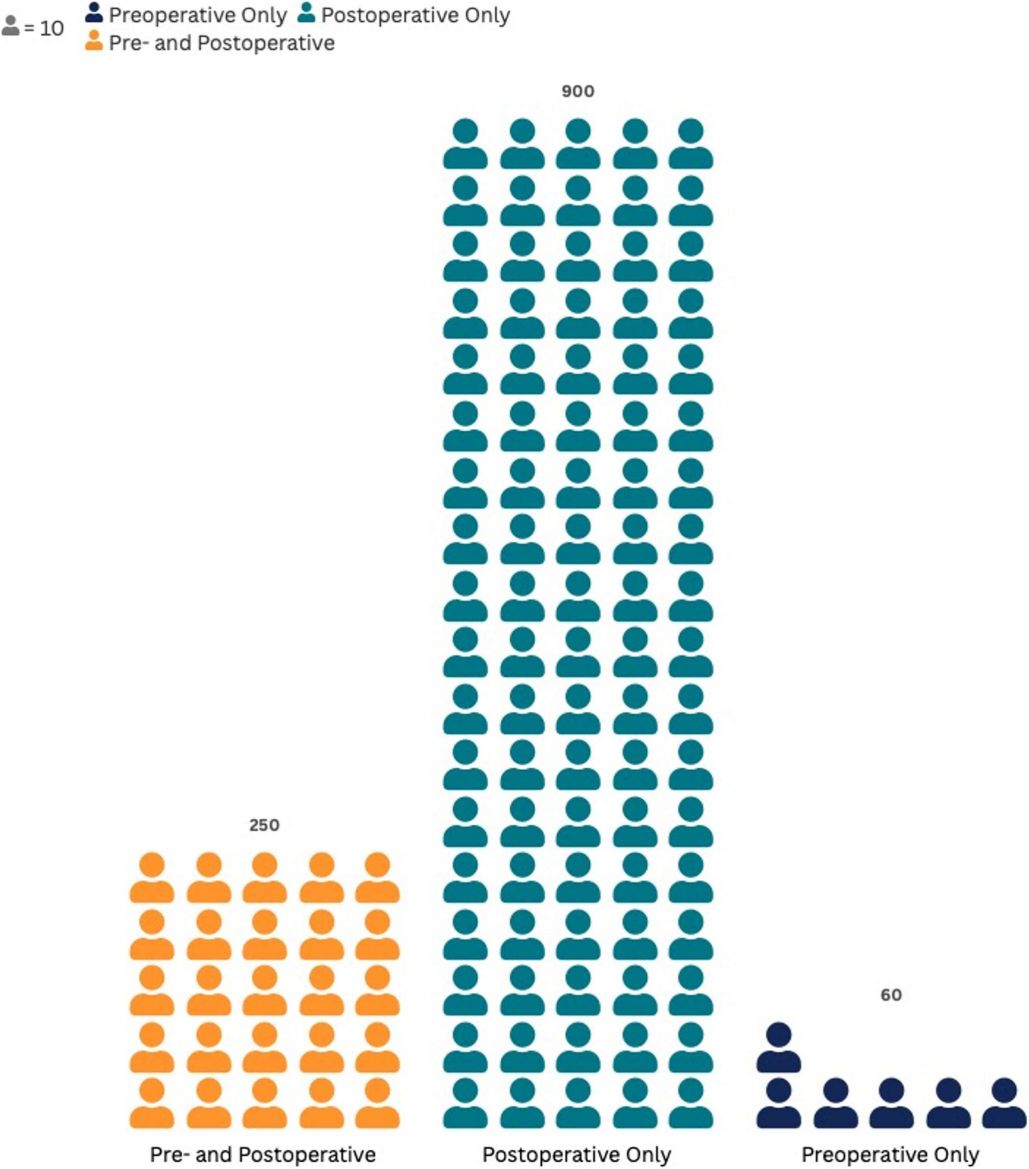
Distribution of antibiotic prescription regimens (preoperative, postoperative, both) reported in coronectomy procedures

The included studies reported substantial heterogeneity in the timing of antibiotic prescription for coronectomy (Supplementary Table 4). Three main patterns were identified: (a) preoperative prophylaxis [5, 20–23], including a prescription of amoxicillin [23, 24] or clindamycin [25], as described in the original reports; (b) postoperative therapy, the most frequent approach, with antibiotics prescribed for 3–7 days, most commonly amoxicillin (500 mg every 8 h) or alternatives such as clindamycin or metronidazole [3, 11, 19, 26–50]; and (c) combined regimens, consisting of a single preoperative dose followed by postoperative administration for 4–7 days [1, 51–60] (details can be observed in Fig. 4 and Supplementary Table 4).

With respect to antibacterial agents, a predominance of beta-lactams was reported, particularly amoxicillin, which is administered alone in different prophylactic or therapeutic regimens [3, 23, 24, 26, 30, 32, 35, 41–45, 47–49, 52, 57, 61]. In several studies, amoxicillin was combined with clavulanic acid, especially in pre- and postoperative protocols lasting 4 to 7 days [29, 36, 46, 50, 53–56, 60]. Penicillin *V* and combinations with ampicillin and cloxacillin were occasionally [1, 39]. Nitroimidazoles (metronidazole) were reported in some studies, either alone or in combination with amoxicillin, usually in short-term regimens of 3 to 5 days [16, 18, 26, 35, 40, 41]. Lincosamides (clindamycin) were described in a considerable number of publications, generally as alternatives for patients allergic to penicillin [3, 13, 32, 45, 47, 60]. Macrolides (azithromycin) were cited in a few cases, as were first-generation cephalosporins (e.g., cefradine, cephalexin), typically in short regimens [40, 59]. Tetracyclines (doxycycline) were identified in only one study [11]. Some protocols described unusual combinations, such as ampicillin, cloxacillin, and metronidazole [39]. Several articles did not specify which antibiotic had been administered to the patients [5, 17, 19–22, 27, 28, 34, 37, 58]. Supplementary Table 4 provides the complete extracted data from the original articles, whereas Table 1 presents an overview of the antibiotic classes, prescribed agents and regimens, and the number of patients included in the studies.

**Table 1:** Antibiotic classes, agents, regimens and patients with prescriptions reported in coronectomy primary studies

Class	Antibiotic	Number of studies*†	Number of patients*†	Typical regimen
Beta-lactams	Amoxicillin (AMX)	25	1200	500 mg q8h for 3–7 days; or 2 g 1 h preoperatively
Beta-lactams	Amoxicillin + Clavulanic Acid (AMX + CLA)	10	400	1 g q8h for 4–5 days; or 2 g 1 h preoperatively
Beta-lactams	Penicillin V/Ampicillin + Cloxacillin	2	80	1 g q6h (5–7 days) or combinations (AMP + CLO+MTZ) ‡
Nitroimidazoles	Metronidazole (MTZ)	8	300	400 mg q8h for 3–5 days
Lincosamides	Clindamycin (CLI)	6	250	300–600 mg/day for 5–7 days
Macrolides	Azithromycin (AZT)	2	50	500 mg/day for 5–6 days
Cephalosporins	Cefradine/Cephalexin (1st gen)	2	60	Short course (3 days)
Tetracyclines	Doxycycline (DXC)	1	30	200 mg day 1, then 100 mg for 7 days
Combinations	AMP + CLO + MTZ‡	1	40	AMP 250 mg + CLO 250 mg + MTZ 400 mg q8h, 5 days

*Totals may exceed the number of studies or patients when more than one antibiotic or regimen was reported within the same study

†In some studies, the specific antibiotic prescribed or the exact number of patients receiving antibiotic was not specified

‡Combination regimen consisting of ampicillin (AMP) + cloxacillin (CLO) + metronidazole (MTZ)

**Fig. 5:**
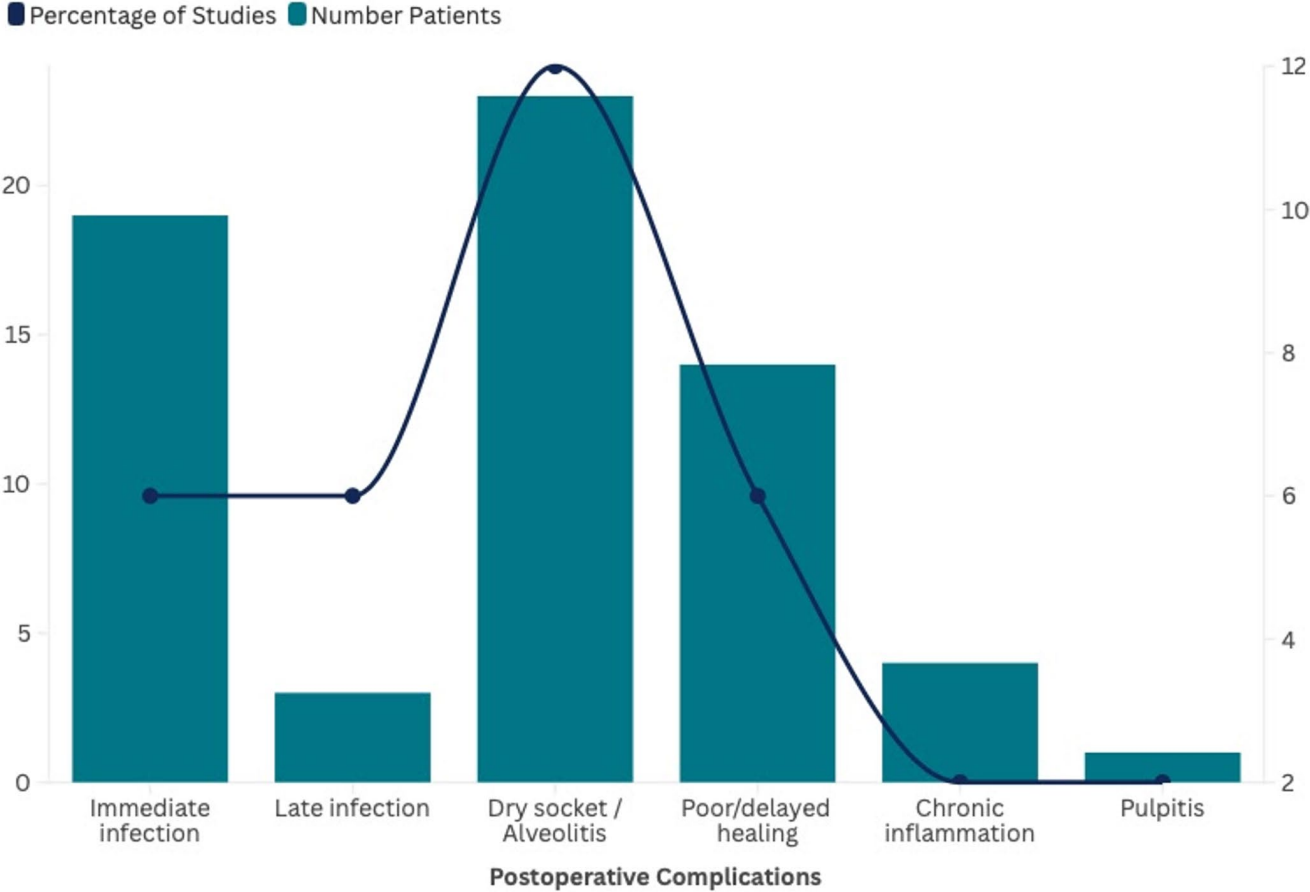
Postoperative complications reported after with coronectomy. The bars represent the number of patients affected by each complication, while the line indicates the percentage of studies that reported them

Despite the use of antibiotics, postoperative infection cases were reported in limited number of studies (twelve), affecting approximately 60 patients (2.82% of the sample), both in the immediate and later stages [3, 17, 19, 29, 32, 33, 38, 43, 45, 49, 51, 53, 56]. Amoxicillin, alone or in combination with clavulanic acid, was the most frequently prescribed antibiotic in these reports, reflecting its overall predominance in coronectomy protocols rather than implying an association with infection occurrence. Other antibiotics, such as clindamycin and metronidazole, were reported. Additional postoperative complications reported after coronectomy included alveolitis/dry socket, delayed or incomplete healing, chronic periodontal disease in adjacent teeth, and less frequent events such as pulpitis and pain (Fig. 5 and Supplementary Table 4).

Concerning the pathologies associated with third molars undergoing coronectomy, the most frequently identified condition described were pericoronitis, periodontal disease, dental caries, and periodontal pockets in adjacent teeth, whereas odontogenic cysts and tumors were observed less often [11, 18, 20, 22, 24–26, 37, 43, 44, 46, 51, 59]. These conditions were described in approximately 300 teeth across 13 articles. Amoxicillin, alone or in combination with clavulanic acid, was the most commonly prescribed antibiotic, consistent with its widespread use across the included studies. Clindamycin and metronidazole were used less frequently. The predominant regimen involved postoperative administration for 3–7 days, although preoperative prophylaxis was also employed in a few studies. Postoperative infection or other complications were reported in a limited subset of these cases [43, 51] (Supplementary Table 4).

With respect to adjuvant therapies associated with coronectomy, six studies cited their use in approximately 200 patients [21, 25, 28, 38, 40, 42, 59]. Most of these interventions, including bone grafting, the use of collagen membranes, fibrin sponges, and platelet-rich fibrin, were described as adjuvant to promote guided bone healing. Only one study [42] investigated ozone therapy as an adjuvant to enhance antibiotic action, which was applied to a subset of 20 out of 130 patients (Supplementary Table 4).

## Discussion

This scoping review was conducted to map and summarize information on the use of antibiotics in the coronectomy of mandibular third molars, as no previous studies with this specific focus were identified in the literature. The results revealed considerable heterogeneity in prescription protocols, which may reflect a combination of factors, including differences in study design, variability in reporting practice, historical prescribing norms, and the absence of standardized guidelines, highlighting differences in institutional practices and clinical decision-making. Although the lack of well-established recommendations for antibiotic use in dental surgical procedures has already been reported in previous research [62, 63], studies addressing this issue remain essential, as they contribute to the broader debate on bacterial resistance, which is currently recognized as one of the greatest global public health challenges [64].

Antibiotic resistance, which compromises therapeutic effectiveness by favoring the selection of resistant microorganisms, arises mainly from the indiscriminate and prolonged use of these drugs [65]. In dentistry, it is estimated that a substantial proportion of prescriptions are unnecessary, particularly in low-risk clinical situations such as simple surgical procedures or cases in which local intervention alone would be sufficient to control the infection process [66]. In the specific context of coronectomy, despite the intentional retention of dental roots within the alveolus, the available literature reports low frequency of postoperative infection or alveolitis following the procedure [1, 6]; however, these findings are descriptively, without implying causality or comparative effectiveness, a consideration that is consistent with the results of the present study.

The analysis of coronectomies performed in third molars with preexisting pathologies aimed to assess whether these conditions would represent a specific indication for antibiotic prescription and, if so, which regimens would be adopted. Given the greater likelihood of infectious or inflammatory processes in such cases, the predominant approach would be the administration of preoperative prophylactic antibiotics. However, even in these situations, the most common practice was the postoperative prescription of amoxicillin for 5–7 days, which appears to reflect a routine clinical pattern rather than a pathology-specific indication. Postoperative infections or other complications were reported in only a limited number of the cases, and no comparative assessment was possible to determine whether the presence of preexisting pathologies influenced infection risk. Moreover, most cases of coronectomy in teeth with pathological conditions have been described in case reports, which limits comparability and restricts the generalizability of findings owing to the methodological limitations inherent to this study design.

Amoxicillin was the most frequently prescribed antibiotic in the included studies, both preoperatively and postoperatively. This finding is consistent with evidence that the oral microbiota is generally sensitive to penicillin, which is considered the first-line antibiotic for the treatment of odontogenic infections [62, 67]. The combination of amoxicillin with clavulanic acid has also been frequently reported. However, studies on odontogenic infections emphasize that this association, while expanding the antimicrobial spectrum, should not be routinely indicated when the microbiota is not known to be resistant to penicillin to minimize the risk of promoting bacterial resistance [62]. Similarly, the American Association of Oral and Maxillofacial Surgeons (AAOMS) guidelines [68] recognize amoxicillin + clavulanic acid as an option in specific clinical situations, particularly when infection by β-lactamase–producing bacteria is suspected, but do not recommend its routine use in dentoalveolar procedures. In cases where amoxicillin was prescribed either pre- or postoperatively, with or without clavulanic acid, infections or other complications such as alveolitis were reported. This observation should be interpreted in light of the overall predominance of this antibiotic in the included protocols, rather than as an indication of reduced clinical effectiveness.

Although some articles did not specify the antibiotic prescribed or the period of administration, mentioning only that an antibacterial drug had been used in patients undergoing the procedure, several other agents, such as clindamycin, cephalosporins, azithromycin, and metronidazole, were reported in the included studies. Metronidazole and clindamycin are primarily employed as alternatives for patients allergic to amoxicillin. Except for metronidazole, which was also administered preoperatively, all other drugs were prescribed during the postoperative period. A recent network meta-analysis evaluating antibiotics use in third molar suggested that the prophylactic was more effective than the use of a placebo in reducing postoperative infection [63], however, this evidence is not directly transferable to coronectomy, which differs technically and biologically from conventional extraction and should be interpreted with caution. Moreover, the authors of that meta-analysis emphasized the high risk of bias and the low quality of the included articles. The studies that used antibiotics other than amoxicillin generally reported a small number of patients with postoperative infections or other complications, precluding any inference regarding comparative effectiveness among agents.

Another noteworthy finding was the use of antibiotic combinations, including ampicillin + cloxacillin + metronidazole [39], cefradine + metronidazole [16], amoxicillin + clindamycin [60], and amoxicillin + doxycycline [11]. According to the AAOMS guidelines [68], antibiotic combinations are not recommended for routine dentoalveolar procedures and should be reserved for specific clinical scenarios requiring a broader antimicrobial spectrum coverage, such as severe or mixed infections. Despite this recommendation, in the studies reporting combination therapy, the third molars did not present preexisting pathologies, nor did the patients have systemic conditions, as these were exclusion criteria in the present review. This finding underscores the importance of critically addressing the indiscriminate use of antibiotic combinations and their implications for the development of bacterial resistance.

Guided bone augmentation was the most frequently reported adjuvant therapy in the studies included in this review. Although antibiotics were prescribed in all these cases, it was not possible to determine whether their use was related specifically to the coronectomy procedure or to the regenerative intervention as systemic antibiotics are commonly prescribed as part of guided bone regeneration [60, 69].

As a limitation of the present study, in addition to those inherent to the methodological approach of a scoping review, it should be noted that articles not reporting the use of antibiotics were excluded. This prevented a direct comparison of postoperative infection rates between coronectomy procedures performed with and without antibiotic prescription, thereby limiting the assessment of the actual need for their prescription in this context. Furthermore, patient-related and tooth-related factors, such as systemic health conditions, age, oral hygiene status, smoking habits, and the anatomical complexity of the impacted tooth, may influence postoperative infection risk and were inconsistently reported across the included studies, further limiting the generalization of the findings. Nevertheless, the infection rate reported after coronectomy have been addressed in a recent systematic review [6], which described similar low frequencies of infection while emphasized the need for caution interpretation due to low level of evidence. In this context, the need for well-designed clinical trials comparing coronectomy procedures performed with and without antibiotic use, is further reinforced.

## Conclusion

This scoping review highlighted the considerable heterogeneity in antibiotic use during coronectomy procedures, with no specific clinical situation consistently linked to its prescription. The predominant pattern was postoperative therapy, most commonly amoxicillin alone or in combination with clavulanic acid. Postoperative infections and other complications were reported across studies; however, the heterogeneity of study designs and outcomes reporting precluded assessment of any consistent association with specific antibiotic regimens or with preoperative pathological conditions of third molars. Overall, the findings reveal a lack of standardization and limited evidence to support specific protocols for antibiotic use in coronectomy. Well-designed studies are still needed to establish clear indications, optimal agents, dosages, and treatment durations to guide clinical practice, highlighting the discussion of antimicrobial resistance and reinforcing the importance of prudent prescribing.

## Supplementary Information

Below is the link to the electronic supplementary material.

Supplementary Material 1

## Data Availability

No datasets were generated or analysed during the current study.
