# Supplementary material for: Prophylactic and postoperative antibiotic therapy for coronectomy procedures in mandibular third molars: mapping the evidence through a scoping review
Source: Oral Maxillofac Surg. 2026 Mar 6;30(1):46. doi: 10.1007/s10006-026-01516-w (PMC12963269; doi:10.1007/s10006-026-01516-w)
Supplement: Supplementary file 1 — Supplementary Material 1 [file 10006_2026_1516_MOESM1_ESM.pdf]

Supplementary Material

Antibiotic therapy for coronectomy procedures in mandibular third molars: Mapping the evidence through a scoping review

Silva LS; Morel LL; Burkert JR; Martos J, Xavier CB; Damian FM
Federal University of Pelotas – melissaferesdamian@gmail.com / melissa.damian@ufpel.edu.br

Supplementary Table 1: Search strategy development for the PubMed/Medline database and adjusted for the other databases

Database	Search strategy
PubMed / Web of Science / Scopus	((coronectomy) OR (coronectomies) OR (third molar coronectomy) OR (third molars coronectomy) OR (coronectomy third molar) OR (wisdom tooth coronectomy) OR (wisdom teeth coronectomy) OR (lower third molar coronectomy) OR (lower third molars coronectomy) OR (coronectomy technique) OR (coronectomya) OR (odontectomy) OR (partial odontectomy) OR (intentional partial odontectomy) OR (intentional root retention) OR (intentional partial root retention))
Embase*	((coronectomy) OR (coronectomies) OR (third molar coronectomy) OR (third molars coronectomy) OR (coronectomy third molar) OR (wisdom tooth coronectomy) OR (wisdom teeth coronectomy) OR (lower third molar coronectomy) OR (lower third molars coronectomy) OR (coronectomy technique) OR (coronectomya) OR (partial odontectomy) OR (intentional partial odontectomy) OR (intentional root retention) OR (intentional partial root retention))
Cochrane Library**	((coronectomy) OR (coronectomies) OR (third molar coronectomy) OR (third molars coronectomy) OR (coronectomy third molar) OR (wisdom tooth coronectomy) OR (wisdom teeth coronectomy) OR (lower third molar coronectomy) OR (lower third molars coronectomy) OR (coronectomy technique) OR (coronectomya) OR (odontectomy) OR (partial odontectomy) OR (intentional partial odontectomy) OR (intentional root retention) OR (intentional partial root retention))
Scielo / Virtual Health Library***	((coronectomy) OR (coronectomies) OR (third molar coronectomy) OR (third molars coronectomy) OR (coronectomy third molar) OR (wisdom tooth coronectomy) OR (wisdom teeth coronectomy) OR (lower third molar coronectomy) OR (lower third molars coronectomy) OR (coronectomy technique) OR (coronectomya) OR (odontectomy) OR (partial odontectomy) OR (intentional partial odontectomy) OR (intentional root retention) OR (intentional partial root retention) OR (coronectomia) OR (odontectomia))

Table Captions: * The term "odontectomy", identified as an *Entree Term* for "tooth extraction", was removed
** Search with a filter for Clinical Trials and Clinical Trials Protocols
*** Descriptors in Health Sciences were added in the search strategy

Supplementary Table 2: Excluded study after full-text analysis with reasons for exclusion (N = 115 registers)

**Authors (Year)**	**Reason 1: no antibiotics prescription / information (n= 60)**	**Reason 2: study design (n= 35)**	**Reason 3: other teeth (n= 3)**	**Reason 4: unintentional coronectomy (n= 1)**	**Reason 5: duplicate sample (n= 5)**	**Reason 6: patients ASA III to VI (n= 2) †**	**Reason 7: Other reasons (n = 9) ‡**
Al-Sarraj et al. (2022)						X	

Alves et al. (2018)						X
Agbaje et al. (2015)	X					
Ahnad (2023)		X				
Ahmed et al. (2011)		X				
Ahmed et al. (2023)	X					
Alqhtani et al. (2023)	X					
Aravindaksha et al. (2015)	X					
Assael (2004)		X				
Arpag (2024)		X				
Becerra (2017)		X				
Biocanin; Todorovic (2014)			X			
Bonte (2015)		X				
Brignardello-Petersen (2018)		X				
Cashman et al. (2017)		X				
Cheung; Leung (2017)	X					
Dallaserra (2020)		X				
De Souza et al. (2020)		X				
Drage; Renton (2002)				X		
Dudak et al. (2025)	X					
Elo; Zakhary (2016)		X				
Escudeiro et al. (2018)			X			
Freedman (1997)	X					
Gady; Fletcher (2014)		X				
Gaikwad et al. (2011)	X					
Garcia-garcia (2005)		X				
Garcia-garcia (2006)		X				
Gazoli et al. (2024)	X					
Geiger et al. (2020)	X					
Geisler (2013)	X					

Gleeson et al. (2012)		X		
Gongora et al. (2019)				X
Goto et al. (2012)	X			
Hatano et al. (2009)	X			
James et al. (2023)	X			
Jing et al. (2023)				X
Kim et al. (2014)	X			
Kouwenberg et al. (2016)	X			
Kumar; Hussian (2019)	X			
Kurita et al. (2015)	X			
Landi et al. (2010a)	X			
Landi et al. (2010b)	X			
Le et al. (2024)	X			
Leung (2015a)	X			
Leung (2015b)		X		
Leung; Cheung (2009)	X			
Leung; Cheung (2012)			X	
Leung; Cheung (2015)	X			
Leung; Cheung (2018)			X	
Leung et al. (2020)	X			
Leung; Pang (2024)		X		
Linares et al. (2015)		X		
Linares et al. (2016)	X			
Malden et al. (2010)	X			
Manor et al. (2016)	X			
Matsushita et al. (2015)	X			
Maulina et al. (2018)				X
Meira et al. (2019)	X			

Mendes et al. (2018)	X			
Middlehurst et al. (1988)	X			
Monaco et al. (2015)			X	
Monaco et al. (2019)			X	
Monaco et al. (2023)			X	
Min et al. (2022)		X		
Mumtaz et al. (2018)	X			
Nowak et al. (2014)	X			
Orentlicher, Horowitz (2022)	X			
Pacci et al. (2014)	X			
Pang; Leung (2022)		X		
Patel et al. (2010)		X		
Patel et al. (2013)		X		
Patel et al. (2014)	X			
Patel et al. (2016)		X		
Patel et al. (2020)	X			
Peat (2017)	X			
Pedersen et al. (2018)	X			
Pedersen et al. (2019)	X			
Pippi (2009)				X
Pogrel (2007)		X		
Pogrel (2009)		X		
Radia et al. (2024)	X			
Rai et al. (2022)				X
Ravi-Shankar (2019)	X			
Rolek, Plawecke (2024)				X
Renton et al. (2005)	X			
Rubio (2010)		X		

Samani et al. (2016)			X	
Schlabe et al. (2017)	X			
Shaath (2019)		X		
Shah et al. (2015)	X			
Shetty; Malli (2017)	X			
Simons et al. (2023)	X			
Simons et al. (2024)	X			
Steel et al. (2022)		X		
Steinberg; Nick (2021)	X			
Szalma; Soós (2019)		X		
Szalma et al. (2022)	X			
TaniguchI et al. (2017)	X			
Tkachenko et al. (2019)				X
Tolstunov et al. (2011)				X
Tuk et al. (2021)				
VignudellI et al. (2018)	X			
Wang et al. (2022)	X			
Wofford; Miller (1987)	X			
Wolf; Dym (2013)		X		
Wong et al. (2015)	X			
Wood (2013)		X		
Wu et al. (2019)				X
Yamamoto-Valenzuela et al. (2022)		X		
Yan et al. (2019)	X			
Yan et al. (2020)	X			
Yan et al. (2020)	X			
Yeung et al. (2018)	X			
Yilmaz (2023)		X		

Zallen; Massoth (2005)	X
Zhang et al. (2023)	X

Table Captions: †ASA classification system according to the American Society of Anesthesiologists.
‡Other reasons for exclusions included (n=9): conventional odontectomy (Gongora et al., 2019; Maulina et al., 2018); exclusively radiographic follow-up data (Pedersen et al., 2019); language – Ukrainian (Tkachenko et al.; 2019) – Chinese (Wu et al.; 2019, Jing et al., 2023); ostectomy technique (Tolstunov et al., 2011); patient refusal of coronectomy (Rai et al., 2022); treatment of pericoronaritis (Rolek, Plawecke, 2024).

Supplementary Table 3: Descriptive data from 50 primary studies included in the scoping review (chronologically arranged)

AUTHOR, YEAR	COUNTRY	LANGUAGE	STUDY DESIGN	PRIMARY OUTCOME	PATIENTS FOLLOW-UP
O'Riordan, 2004 [17]	England	English	Case Series / Retrospective	To evaluate the infection rate of retained roots after coronectomy	120 months
Pogrel et al, 2004 [5]	USA	English	Case Series / Retrospective	To evaluate coronectomy as a surgical alternative to reduce damage to the IAN	42 months
Dolanmaz et al., 2009 [27]	Turkey	English	Case Series / Retrospective	To evaluate the effectiveness of coronectomy	48 months
Recio Lora et al., 2009 [20]	Spain	Spanish and English	Case Report	To report a clinical case of coronectomy	12 months
Sencimen et al., 2010 [28]	Turkey	English	Observational Study / Case-control Prospective	To evaluate the need to associate endodontic treatment in coronectomies	12 months
Cilasun, 2011 [29]	Turkey	English	Case Series / Retrospective	To evaluate the success of coronectomy compared to conventional extraction	6 to 29 months
Monaco et al., 2012 [52]	Italy	English	Observational Study / Prospective	To evaluate postoperative complications of coronectomies	12 months
Deboni et al., 2013 [30]	Brazil	Portuguese	Case Report	To describe radiographic and tomography diagnoses, coronectomy technical details and postoperative follow-up	3 months
Leizerovitz, Leizerovitz, 2013 [25]	USA	English	Case Report	To present the Modified and Grafted Coronectomy Technique, and to describe the measures to prevent or minimize the known drawbacks of standard coronectomy	36 months

Patel et al., 2013 [26]	United Kingdom	English	Case Series / Retrospective	To evaluate results after coronectomy of teeth with dentigerous cysts	40 months
Rocha; Oliveira, 2014 [31]	Brazil	English	Case Report	To report a clinical case of coronectomy	24 months
Dias-Ribeiro et al., 2015 [53]	Brazil	Portuguese	Case Report	To assessment of neurosensory deficit, postoperative infection, and the effectiveness of the coronectomy surgical technique	24 months
Frenkel et al., 2015 [32]	Israel	English	Case Series / Retrospective	To evaluate the success rate of coronectomies and, in case of failure, evaluate reinterventions	12 months
Kohara et al., 2015 [33]	Japan	English	Case Series / Retrospective	To investigate the morbidity of coronectomy and to monitor the behavior and migration pattern of the retained roots postoperatively	36 months
Monaco et al., 2015 [54]	Italy	English	Observational Study / Cohort Prospective	To evaluate postoperative complications of coronectomies	24 months
Vignudelli et al., 2015 [55]	Italy	English	Case Report	To report a clinical case of coronectomy with histological analysis of the roots	18 months
Guerrero et al., 2016 [34]	Mexico	Spanish	Case Series / Retrospective	To evaluate clinically the use of coronectomy as an alternative to minimally invasive treatment to avoid the lesion of the IAN	6 months
Mukherjee et al., 2016 [35]	India	English	Clinical Study / Prospective	To evaluate the outcomes related to retained roots after coronectomy	24 months
Elo et al., 2017 [21]	USA	English	Case Series / Retrospective	To report the long-term results of coronectomy associated with bone grafting	60 months
Franco et al., 2017 [56]	Italy	English	Case Series / Retrospective	To describe postoperative morbidity related to secondary intention healing after coronectomies	24 months
Henien et al., 2017 [18]	England	English	Case Series / Retrospective	To evaluate cases of coronectomy in teeth with dentigerous cysts	120 months
Kim et al., 2017 [36]	South Korea	English	Case Series / Retrospective	To examine the fate and complications of residual roots by long-term follow-ups and to inform whether coronectomy could be suggested as an alternative treatment	61.7(±27.8) months
Naji et al., 2017 [37]	Morocco	English	Case Report	To report a case of coronectomy associated with pericoronitis	36 months

Vignudelli et al., 2017 [57]	Italy	English	Observational Study / Cohort Prospective	To analyze periodontal healing after coronectomy	9 months
Leung, 2018 [38]	Hong Kong	English	Randomized Clinical Trial / Prospective	To compare the root migration rate and surgical morbidities after coronectomy with or without adjunctive guided bone regeneration	24 months
Rosa et al., 2018 [58]	Brazil	Portuguese	Case Series / Retrospective	To evaluate trans- and postoperative complications, and the root migration index, in coronectomy	6 to 49 months
Singh et al., 2018 [39]	India	English	Randomized Clinical Study / Prospective	To compare postoperative complications of coronectomy and conventional extraction	6 months
Kang et al., 2019 [40]	China	English	Clinical Study / Prospective	To compare near-term outcomes between coronectomy and traditional extraction, and evaluate long-term complications after coronectomy	36 months
Pellat et al., 2019 [22]	England	English	Case Report	To report a severe case of infection after coronectomy	2 months
Pitros et al., 2019 [19]	German	English	Observational Study / Cohort Retrospective	To indicate the short- and long-term outcomes of coronectomy	120 months
Shokouhi et al., 2019 [41]	England	English	Case Series / Prospective	To analyze histologically the roots removed after coronectomies	-
Cosola et al., 2020 [42]	Italy and South Korea	English	Case Series / Retrospective	To repord data and build indication guidelines on coronectomy	36 to 84 months
Mendes et al., 2020 [23]	Brazil	English	Case Series / Prospective	To report a series of coronectomy cases	12 months
Moura et al., 2020 [43]	Brazil	English	Case Series / Retrospective	To present outcomes related to coronectomy	24 months
Moura Batista et al., 2020 [44]	Brazil	Portuguese	Case Report	To evaluate the surgical technique and possible complications of coronectomy	6 months
Sureshkannan et al., 2020 [45]	Emirates	English	Pilot Study / Prospective	To evaluate the use of coronectomy to prevent sensorineural deficit to the IAN	12 months
Lee et al., 2021 [59]	South Korea	English	Case Series / Retrospective	To analyze root migration and its influencing factors after coronectomy	6 months
Maheshwari et al., 2021 [16]	Pakistan	English	Observational Study / Cross-sectional Prospective	To compare the results of coronectomy and conventional extraction	1.5 months
Mariano et al., 2021 [60]	Brazil	English	Case Report	To report a clinical case of coronectomy associated with the use of fibrin-rich plasma	12 months

Braimah et al., 2022 [46]	Saudi Arabia	English	Observational / Retrospective	To evaluate sociodemographic factors and sequels of coronectomy	12 months
Lamiae et al., 2022 [24]	Morocco	English	Case Series / Retrospective	To report a case series of coronectomy	24 months
Pinto et al., 2022 [47]	Brazil	English	Observational / Prospective	To evaluate the root migration after coronectomy and associate factors with this outcome	12 months
James et al., 2023 [48]	Nigeria	English	Randomized Controlled Clinical Trial / Prospective	To compare neurosensory deficits of IAN using one-stage complete extraction with the two-stage partial coronectomy technique	12 months
Hamad, 2024 [3]	Iraq	English	Observational / Prospective	To compare surgical complications and neurosensory deficits after coronectomy and complete removal of mandibular third molars	24 months
Pang et al., 2024 [49]	China	English	Randomized Controlled Trial / Prospective	To compare the periodontal healing at the distal of the adjacent second molar after coronectomy or surgical removal and the surgical morbidities of the two techniques	6 months
Salgado-Peralvo et al., 2024 [61]	Spain	English	Case Report	To present two clinical cases in which coronectomy was performed given the associated high risk of damage to the IAN and mandibular fracture	12 months
Haskan et al., 2025 [51]	Turkey	English	Observational Cohort Study / Prospective	To evaluate the development of endodontic lesion in coronectomy procedures	6 months
Tamer et al., 2024 [50]	Turkey	English	Observational / Retrospective	To evaluate long-term (5 years) clinical and radiologic outcomes after mandibular third molar coronectomy	60 months
Sharif et al., 2025 [1]	Norway	English	Observational / Retrospective	To analyze the outcomes of coronectomy, the use of antibiotics, and associated post-operative complications	12 months
Van Bodegraven et al., 2025 [11]	Netherlands	English	Observational Cohort Study / Prospective	To investigate the safety of coronectomy teeth with dental caries and dentigerous cysts	12 months

Table Captions: USA = United States of America; IAN = Inferior Alveolar Nerve

Supplementary Table 4: Sample of patients and mandibular third molars treated with coronectomy, associated adjuvant therapies, mandibular third molar preoperative pathologies, antibiotics prescription, occurrence of immediate / late postoperative infection (period occurrence), and other complications (period of occurrence), collected in primary studies (50 registers – chronologically arranged)

Author, Year	Sample Patients And Teeth	Age of Patents Sample	Adjuvant Therapy Applied (Patients Sample)	Third Molars Preoperative Pathologies Reported (Patients / Teeth Sample)	Preoperative Antibiotics	Postoperative Antibiotics	Immediate Postoperative Infection Reported (Sample)	Late Postoperative Infection Reported (Sample / Follow-up period)	Late Postoperative Complication Reported (Type / Sample / Follow-up period)
O'Riordan, 2004 [17]	52 patients<br>-	-	-	-	-	NR	1 patient	3 patients<br>(18 moths to 7 year)	Dry sockets (immediate postoperative period)<br>Chronic advanced periodontal disease (until roots removed - 7 years later)
Pogrel et al., 2004 [5]	41 patients 50 teeth	-	-	-	NR	-	-	-	-
Dolanmaz et al., 2009 [27]	43 patients<br>(M:20 / W:23)<br>47 teeth	-<br>Range: 18 to 38 years	-	-	-	NR	-	-	-
Recio Lora et al., 2009 [20]	1 patient (M:1)<br>1 tooth (38:1)	25 years<br>-	-	Pericoronitis<br>(1 patient)	NR	-	-	-	-
Sencimen et al., 2010 [28]	10 patients (M:4 / W:6)<br>16 teeth	M:23 years / W:19 years<br>-	Endodontic treatment<br>(5 patients)	-	-	NR	7 teeth on group coronectomy + endodontic<br>7 teeth on group coronectomy	-	-
Cilasun et al., 2011 [29]	-<br>88 teeth	Mean: 27.19 years	-	-	-	625mg AMX CLA (2 x 1) for 5 days	-	1 patient<br>(3 to 4 weeks)	-
Monaco et al., 2012 [52]	37 patients<br>(M:17 / W:20)<br>43 teeth	Mean: 31 (±2) years<br>-	-	Pericoronitis and Periodontal disease<br>(37 patients)	2g AMX (1h before surgery)	1g AMX (every 8h)<br>for 4 days	-	-	Alveolitis<br>(1 patient / 15 days)
Deboni et al., 2013 [30]	1 patient (W:1)<br>1 tooth (38:1)	50 years<br>-	-	-	-	500mg AMX (every 8h)<br>for 7days	-	-	-

Leizerovitz; Leizerovitz, 2013 [25]	1 patient (W:1)<br>2 teeth (38:1 / 48:1)	37 years<br>-	Bone Graft (1 patient)	Infection and periodontal pocket in the adjacent 2M (1 patient)	150mg CLI (40 tablets)	-	-	-	-
Patel et al., 2013 [26]	20 patients<br>20 teeth (38:9 / 48:11)	-<br>Range: 20 to 76 years	-	Dentigerous cist (19 patients)	-	250mg AMX (every 8h) or MTZ (every 8h)	2 patients	-	-
Rocha; Oliveira, 2014 [31]	1 patient (M:1)<br>1 tooth (38:1)	26 years<br>-	-	-	-	500mg AZT (once a day) for 5 days	-	-	-
Dias-Ribeiro et al., 2015 [53]	2 patients (W:2)<br>3 teeth (38:1 / 48: 2)	25 years<br>Range: 24 to 26 years	-	-	2g AMX (1h before surgery)	500mg AMX (every 8h) for 7 days	-	-	-
Frenkel et al., 2015 [32]	173 patients<br>185 teeth	-<br>Range: 17 to 75 years	-	-	-	1.5g AMX or 600mg/d CLI for 7 days	3 patients	-	Unsatisfactory healing (6 patients / 6 months)
Kohara et al., 2015 [33]	92 patients (M:29 / W:63)<br>111 teeth	33.8 years<br>-	-	-	-	CLP for 3 days	-	-	Dry socket (1 patient / 1 month)<br>Incomplete wound closure (7 patients / 3 to 24 months)
Monaco et al., 2015 [54]	94 patients (M:37 / W:57)<br>116 teeth	28.9 (±8.9) years<br>Range: 17 to 56 years	-	-	2g AMX and CLA (1h before surgery)	1g AMX and CLA (every 8h) for 4 days	-	-	Alveolitis (5 patients / 1 month)<br>Pulpitis (1 patient / 6 months)
Vignudelli et al., 2015 [55]	1 patient (M:1)<br>1 tooth (38:1)	44 years<br>-	-	-	2g AMX and CLA (1h before surgery	1g AMX and CLA (every 8h) for 4 days	-	-	-
Guerrero et al., 2016 [34]	30 patients (M:18 / W:12)	-	-	-	-	NR	-	-	-
Mukherjee et al., 2016 [35]	18 patients (M:13 / W:5)<br>20 teeth	27.6 years<br>-	-	-	-	500mg AMX or 400mg MTZ (every 8h) for 3 days	-	-	-
Elo et al., 2017 [21]	78 patients (M:37 / W:41)<br>92 teeth	29.8 (±9.7) years	Bone graft (78 patients)	-	NR	-	-	-	-

		Range: 20 to 59 years							
Franco et al., 2017 [56]	10 patients (M:4 / W:6) -	28 years Range: 18 to 43 years	-	-	2g AMX and CLA (1h before surgery	1g AMX and CLA (every 8h) for 4 days	-	-	-
Henien et al., 2017 [18]	68 patients (M:34 / W:34) 68 teeth	44 years Range: 18 to 84 years	-	Dentigerous cist (68 patients)	-	AMX or MTZ for 5 days	-	-	-
Kim et al., 2017 [36]	7 patients (M:4 / W:3) 9 teeth (38:5 / 48:4)	39.1 (±11.6) years -	-	-	-	AMX and CLA	-	-	-
Naji et al., 2017 [37]	1 patient (W:1) 1 tooth (48:1)	34 years -	-	Pericoronitis (1 patient)	-	NR	-	-	-
Vignudelli et al., 2017 [57]	30 patients (M:9 / W:21) 34 teeth	28 (±7) years Range: 17 to 56 years	-	-	2g AMX and CLA (1h before surgery)	1g AMX and CLA (every 8h) for 4 days	-	1 patient (8 months)	-
Leung, 2018 [38]**	48 patients (M:19 / W:29) 96 teeth (38:48 / 48:48)	26.6 (±5.8) years Range: 19 to 45 years	GBR** (48 patients / 48 teeth)	-	-	375mg AMX and CLA for 5 days	-	1 patient (GBR group) (3 months)	-
Rosa et al., 2018 [58]	15 patients (M:12 / W:3) 19 teeth	38 years Range: 19 to 57 years	-	-	2g AMX	500mg AMX (every 8h) for 5 days	-	-	-
Singh et al., 2018 [39]	15 patients (M:5 / W:10) -	24.9 (±3.9) years -	-	-	-	250 AMP + 250mg CLO + 400mg MTZ (every 8h)	-	-	-
Kang et al., 2019 [40]	55 patients (M:21 / W:34) 55 teeth	26.5 years Range: 18 to 36 years	Resorbable gelatin sponge (55 patients)	-	-	CER and MTZ for 3 days	-	-	Alveolar osteitis (1 patient)
Pellat et al., 2019 [22]	1 patient (M:1) 1 tooth (38:1)	65 years -	-	Periodontal pocket and Infection (1 patient)	NR	-	-	-	-
Pitros et al., 2019 [19]	124 patients (M:33 / W:91) 133 teeth (38:70 / 48:63)	34.3 years -	-	-	-	NR	16 patients	3 patients (> 3 months)	Chronic inflammation (3 patients / > 3 months)
Shokouhi et al., 2019 [41]	80 patients (M:18 / W:62)	31.6 years	-	-	-	500mg AMX or 400mg MTZ	-	1 patient	-

	92 teeth (38:46 / 48:46)	Range: 19 to 70 years				for 5 days			
Cosola et al., 2020 [42]	130 patients (M:64 / W:66) 130 teeth	27.5 (±3.1) years Range: 24 to 34 years	Ozone therapy (Italy center) (20 patients)	-	-	625mg AMX (Korea center) 875mg AMX (Italy center)	-	-	-
Mendes et al., 2020 [23]	21 patients (M:5 / W:16) 35 teeth (38:18 / 48:17)	24.3 (±4.9) years -	-	-	1g AMX (1h before surgery)	-	-	-	-
Moura et al., 2020 [43]	4 patients (W:4) 4 teeth (38:1 / 48:3)	32 years -	-	Pericoronitis (1 patient)	-	500mg AMX (every 8h) for 7 days	-	1 patient (30 days)	Incomplete heling (1 patient / 6 months)
Moura Batista et al., 2020 [44]	1 patient (M:1) 1 tooth (48:1)	24 years -	-	Pericoronitis (1 patient)	-	500mg AMX (every 8h)	-	-	-
Sureshkannan et al., 2020 [45]	45 patients (M:5 / W:40) -	- Range: 24 to 25 years	-	-	-	50mg AMX or 300mg CLI (every 8h) for 3 days	-	-	Dry socket (2 patients)
Lee et al., 2021 [59]	33 patients (M:11 / W:22) 33 teeth	27.1 (±6.2) years Range: 20 to 47 years	-	-	NR	NR	-	-	-
Maheshwari et al., 2021 [16]	18 patients (M:9 / W 9)	25 years -	-	-	-	500mg AMX + 400mg MTZ (every 8h) for 3 days	-	-	-
Mariano et al., 2021 [60]	1 patient (M:1) 1 tooth (48:1)	20 years -	Platelet-rich fibrin (1 patient)	Pericoronitis (1 patient)	500mg AZT	500mg AZT for 6 days	-	-	-
Braimah et al., 2022 [46]	73 patients (M:24 / W:49) 73 teeth (38:28 / 48:45)	28.2 (±6.7) years -	-	Pericoronitis (31 patients) Caries (28 patients)	-	625mg AMX and CLA for 3 days	-	-	-
Lamiae et al., 2022 [24]	9 patients (M:4 / W:5) 12 teeth (38:7 /48:5)	32.5 years Range: 18 to 70	-	Pericoronitis (1 patient) Pulpitis (1 patient) Keratocyste (1 patient)	2g AMX (1h before surgery)	-	1 patient	-	-

				Dentigerous cyst<br>(1 patient)<br>Odontogenic Fibroma<br>(1 patient)					
Pinto et al., 2022 [47]	22 patients<br>(M:17 / W:5)<br>31 teeth (38:14 / 48:17)	27 (±7.8) years<br>Range: 19 to 52 years	-	-	-	500mg AMX (every 8h) or CLI (every 6h)	-	-	-
James et al., 2023 [48]	34 patients<br>(M:16 / W:18)	27.2 (±6,7) years<br>Range: 18 to 51 years	-	-	-	500mg AMX (every 8h) for 5 days	-	-	-
Hamad, 2024 [3]	243 patients<br>(M:88 / W:124)<br>220 teeth	31.7 (±11.3) years<br>-	-	-	-	500mg AMX (every 8h) or CLI 300 mg (every 8h)	14 patients	-	Blending (1 patient / immediate postoperative period)<br>Dray socket (10 patients / immediate postoperative period)<br>Periapical infection (14 patient)
Pang et al., 2024 [49]	40 patients<br>(M:22 / W:18)<br>40 teeth (38:19 / 48:21)	26.7 (±4.6) years<br>Range: 19.5 to 38.7 years	-	-	-	250mg AMX (every 8h) for 5 days	-	-	-
Salgado-Pervalvo, 2024 [61]	2 patients (W:2)<br>2 teeth (38:1 / 48:1)	-	-	-	500 AMX + 125mg CLA (1h before surgery) (1)<br>875 AMX + 125 CLI (1h before surgery) (2)	500 AMX + 125mg CLA for 6 days (1)<br>875 AMX + 125 CLI for 6 days second surgery (2)		-	Persistent pain (1 patient / 15 days)
Haskan et al., 2025 [50]	73 patients<br>(M:34 / W:39)<br>-	Range: 18 to 55 years	-	-	-	125mg CLA + 875mg AMX (every 12h)	-	-	-

Tamer et al., 2024 [51]	68 patients (M:20 / W:48) 75 teeth (38:40 / 48:35)	26.8 (±8.1) years Range: 17 to 41 years	-	-	-	500mg AMX (every 8h) for 5 days	-	-	-
Sharif et al., 2025 [1]	44 patients (M:20 / W:24) 46 teeth (38:22 / 48:24)	28.5 years Range: 18 to 55 years	-	-	1g PEN (every 6h) for 5 to 7 days – only 6 patients	1g PEN (every 6h) for 5 to 7 days – only 2 patients	2 patients	-	-
von Bondegraven et al., 2025 [11]	115 patients (M:66 / W:49) 121 teeth	44.4 (±18.9) years Range: 18 to 91 years	-	Cist (63 teeth) Carie (58 teeth)	-	500mg AMX (every 8h) for 5 days 200mg DXC (first day) / 100mg DXC (plus 7 days)	-	-	-

Table captions: M= man; W= woman; SD= standard deviation; d= day; h= hours; min= minutes; mg= milligrams; g= grams, 2M= second molar, AMX= amoxicillin, MTZ= metronidazole, CLA= clavulanic acid/clavulanate, CLI= clindamycin, CER= cefradine, CLO= cloxacillin, AMP= ampicillin, AZT= azithromycin, CLP= cephalosporin, PEN= penicillin, DXC= doxycycline, NR= not reported (authors did not report the antibiotic used and the drug posology).

* Postoperative outcomes and complications not related to use of antibiotics were not collected.

** Leung (2018) - Guided Bone Regeneration (GBR): Bovine bone + resorbable bilayer collagen membrane.
